# Optically switched multiband antenna based on Vivaldi structure

**DOI:** 10.1038/s41598-022-19813-1

**Published:** 2022-09-19

**Authors:** Peiying Lin, Yuting Wu, Zhouyi Wu, Ruofan Zhuo, Jiangtao Huangfu

**Affiliations:** Laboratory of Applied Research On Electromagnetics (ARE), College of Information Science and Electronic Engineering, Zhejiang University, Hangzhou, 310027 China

**Keywords:** Electrical and electronic engineering, Optoelectronic devices and components

## Abstract

In this study, an optically frequency-reconfigurable antenna with multiband characteristics is proposed utilizing photodiodes. It is developed on the basis of a Vivaldi antenna structure, while the composite radiation structure is realized by introducing three parallel branches in the antenna slot. Three photodiodes on the branches function as photoconductive switches to make the antenna reconfigurable at multiple low frequencies and stable at high frequencies. When the illumination irradiates different photodiodes, the proposed antenna is capable to switch between three narrowband modes, including 300 MHz, 677 MHz, and 1.02 GHz. The radiation gain is measured to reach 0.91 dB, 1.69 dB, 2.96 dB, respectively, while the variation in illumination states is 6.82 dB, 9.93 dB, 17.13 dB, respectively. Meanwhile, this antenna can continue to work stably at 3.2–3.8 GHz and 5.1–6.5 GHz regardless of illumination, with the maximum gain of 7.51 dB. Both simulation and experimental results substantiate the feasibility of the proposed design. This antenna design can transmit and shield the signal of specific frequency with optical control, and has good working characteristics at both high and low frequencies. In the future, it has promising application potential of communication and radar integration.

## Introduction

In recent years, the development of wireless communication and radar systems has triggered a strong demand for integrating multiple functions into a single antenna in such settings as mobile communication and wireless sensing, which would require the automatic conversion between different antenna resonant frequencies. To meet this requirement, there have been a variety of frequency reconfigurable antennas proposed, such as electronically tunable antenna. Diodes are often employed in the electronically tunable antennas controlled by DC current^1–8^. For example, literature^8^ uses two PIN diodes to shift the operating bands under different switching states, and the corresponding bandwidth also changes. However, it is inevitable for complex DC connection and electromagnetic coupling interference to occur. In general, mechanically tunable antennas change the antenna structures partially, such as air-gap height^9^, slab locations^10^ or liquid thickness of an actuator^11^, etc. Magnetic control is considered another feasible method where the operating frequencies of the antenna can be adjusted by changing the bias magnetic field^12^.

The Vivaldi antenna has a promising prospect for wideband-narrowband reconfiguration. Since most of the current on the Vivaldi antenna flows at the very edge of the tapered profile, the disturbance of the surface currents distribution is what the principle of the reconfigurable Vivaldi antennas is based on^13^. The most common implementation method is to apply resonators as filters in different parts of the antenna^14–17^. A reconfigurable Vivaldi antenna with switched resonators is presented in^14^, which has the ability to switch between wideband and multi-narrow bands. In^17^, two pairs of resonators controlled by DC biased PIN diodes are also used to enable a shift from broadband to narrowband. However, it is necessary to add these structure designs with DC connection. Otherwise, Vivaldi antenna can be dynamically reconfigured using such special materials as graphene^18,19^. The graphene-based tunable resistor under DC bias is integrated with multiple modified Vivaldi antennas in^18^ so as to figure out the pattern of dynamical gain manipulation and reconfigurable radiation. In addition, reconfiguration can also be achieved by introducing ionized water fluid switch into the back-slot of a Vivaldi antenna, as demonstrated in^20^. In order to avoid the possibility of DC connection, mechanical control and material update, the method of optically controlled frequency is introduced into reconfigurable antenna design. In contrast, optical tuning antenna can be remotely controlled without direct contact.

The requirements of optically tunable antennas can be met by the use of such semiconductor materials as the silicon switches that are integrated in different ways^21–27^. Their conductivity can be dynamically adjusted based on illumination at certain wavelengths of light. For example, the gain variation of 5.12 dB is achieved by placing two silicon photo switches in the antenna gap for a 212-mW incident laser power^21^. Literature^23^ demonstrates that optically induced conductivity in silicon is a viable tuning methodology for antennas through the addition of small silicon bridging pieces to a standard slot antenna. In^24^, a design for optically switching the resonant frequency of a microstrip patch antenna is proposed through the silicon switches connected to microstrip-line segments of varying lengths. There are two silicon photoconductive switches soldered to the waveguide body of a slotted-waveguide antenna array in^25^ to enable the reconfiguration of frequency tunability and radiation pattern through the frequency bands of 28 GHz and 38 GHz. In^26^, organic semiconductor materials are directly used to replace the traditional metal patch and adjust the resonant frequency of the antenna by means of external illumination. Besides, the photodiodes as mentioned in^28^ are applied as photoconductive switches in antenna design for the transformation of maximum radiation direction in the radiation patterns under two different illumination conditions. In^29^ a reconfigurable antenna is designed using photodiode as optical switching to achieve 3–7 MHz frequency shift. In the research works as mentioned above, however, the number of reconfigured bands and gain variation are limited, which makes it difficult to achieve the goal of independent adjustment of multiple frequency bands.

In this paper, an optically frequency reconfigurable antenna is proposed on the basis of Vivaldi structure. It has multiple parallel branches containing photodiodes coupled to different parts across the tapered slot of the radiating region for the reconfiguration of frequency bands. These photodiodes function as photoconductive switches activated by illumination. The proposed antenna is capable to switch between three narrowband modes, including 300 MHz, 677 MHz, 1.02 GHz. The principle is that light affects the impedance change of photodiodes at different parts, thus forming different resonant loops. Its multiple narrowband frequencies are associated with the number and placement of employed photodiodes under different illumination conditions.

This design enables the small-size Vivaldi antenna to work efficiently at low frequencies. The frequency switching is remotely controlled by light source without DC interference of electric control mode, which simplifies control network. At the same time, three parallel branches introduced into the Vivaldi antenna slot have the effect of decoupling low and high frequency radiation characteristics. The photodiodes cause no disturbance in antenna performance on high frequency bands, so that the high-frequency performance of Vivaldi antenna still maintains good working characteristics corresponding to the size. The proposed antenna can be applied in both communication and radar, which is conducive to the realization of integrated communication and radar system.

In section “Antenna design”, the design of the frequency reconfigurable Vivaldi antenna is elaborated on, while the working principle and equivalent circuits are analyzed in detail. The simulated and experimental results obtained for this optical antenna are presented in sections “Simulation” and  “Measurement results”, respectively. Finally, the conclusions are indicated in section “Conclusion”.

## Antenna design

**Figure 1:**
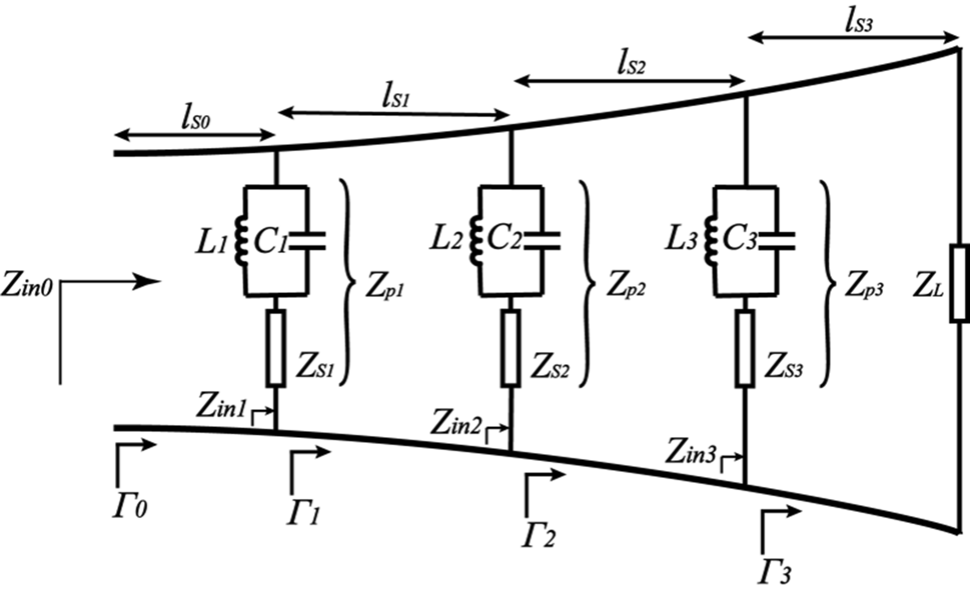
Equivalent transmission line of the reconfigurable Vivaldi antenna.

As a member of the class of aperiodic continuously scaled antenna structures, the Vivaldi antenna in theory has unlimited instantaneous frequency bandwidth, significant gain, linear polarization and simple structure^30^. The curve chosen for a Vivaldi antenna is the exponential expansion expressed as follows:

1 $$\begin{array}{*{20}c} {y = \pm \,\,Ae^{Pz} } \\ \end{array}$$

where *y* represents the half separation distance and *z* refers to the length parameter^30^. In case of multiple parallel branches getting introduced at different parts of the tapered slot in the Vivaldi antenna, the additional resonant current circuit loops with corresponding radiation frequencies can be formed except for the original working frequency of Vivaldi antenna. With the tapered slot equated to a transmission line, Fig. 1 shows that three parallel branches form three external equivalent loads. The overall reflection coefficient $\Gamma_{n}$ along the tapered slot can be expressed as the impedance characteristics of multi segment equivalent transmission line as constructed by slots, where $Z_{sn}$, $Z_{pn}$, $Z_{cm}$, $Z_{inm}$ denote circuit loss and radiation loss, the total impedance of parallel branches, the characteristic resistance of the curve at parallel branches, and the input impedance at different parts, respectively. $Z_{L}$ represents the impedance at the end of the tapered transmission line. Their relation is illustrated below. The following equations are obtained using the analytical method adopted for tapered transmission line^31^:

2 $$Z_{pn} = \frac{{Z_{sn} - \omega^{2} L_{n} C_{n} Z_{sn} + \omega L_{n} j}}{{1 - \omega^{2} L_{n} C_{n} }},n = 1,2,3$$

3 $$\Gamma_{3} = \frac{{\ln \left( {Z_{L} /Z_{c3} } \right)}}{2}e^{{ - j\beta l_{s3} }} \frac{{sin\left( {\beta l_{s3} } \right)}}{{\beta l_{s3} }}$$

4 $$Z_{inm} = Z_{cm} \frac{{1 + \Gamma_{m} }}{{1 - \Gamma_{m} }},m = 0,1,2,3$$

5 $$\Gamma_{2} = \frac{{\ln \left( {Z_{p3} ||Z_{in3} /Z_{c2} } \right)}}{2}e^{{ - j\beta l_{s2} }} \frac{{sin\left( {\beta l_{s2} } \right)}}{{\beta l_{s2} }}$$

6 $$\Gamma_{1} = \frac{{\ln \left( {Z_{p2} ||Z_{in2} /Z_{c1} } \right)}}{2}e^{{ - j\beta l_{s1} }} \frac{{sin\left( {\beta l_{s1} } \right)}}{{\beta l_{s1} }}$$

7 $$\Gamma_{0} = \frac{{\ln \left( {Z_{p1} ||Z_{in1} /Z_{c0} } \right)}}{2}e^{{ - j\beta l_{s0} }} \frac{{sin\left( {\beta l_{s0} } \right)}}{{\beta l_{s0} }}$$

where $\beta = 2\pi /\lambda$, *L* represents the total taper length and $l_{sn}$ refers to the distance of each segment. The resonant frequency of the reconfigurable antenna can be evaluated using the reflection coefficient obtained as input.

**Figure 2:**
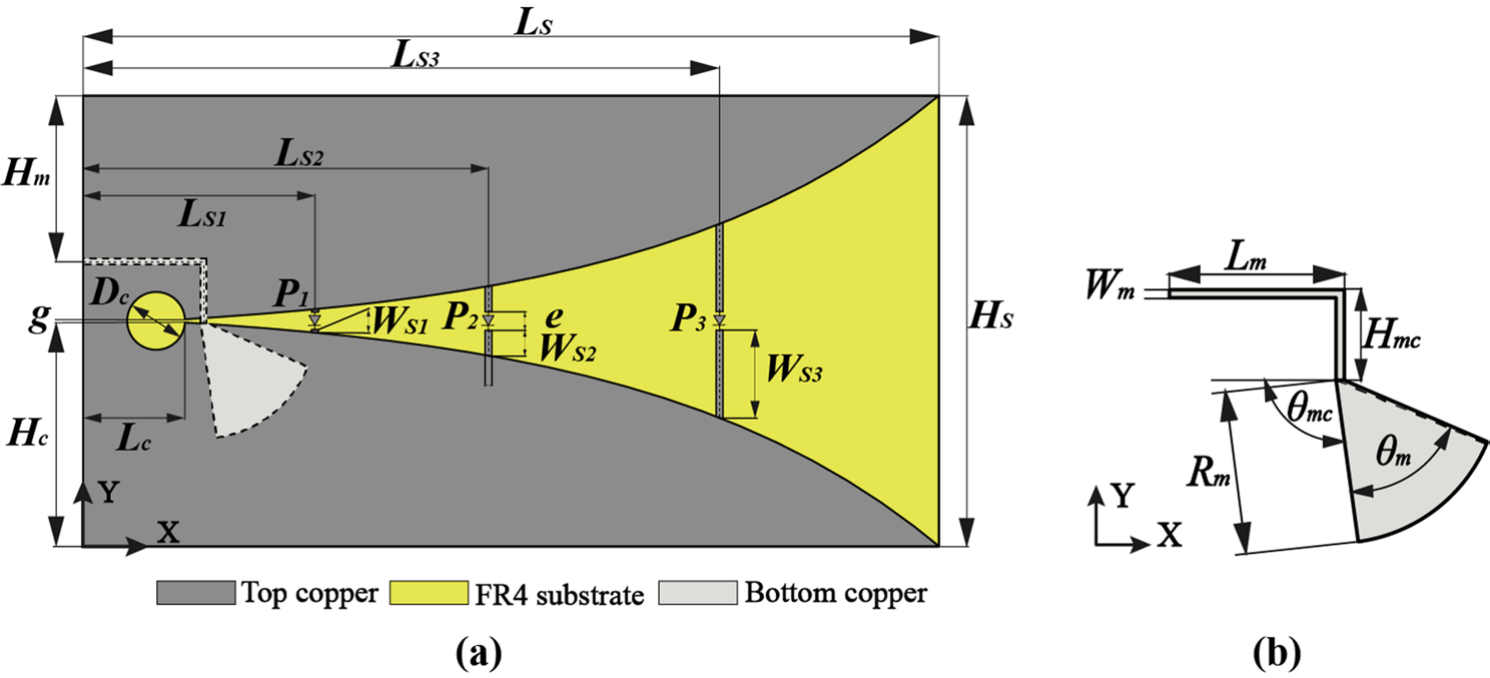
Scheme of the optical Vivaldi antenna with its main parameters: (**a**) global view; (**b**) the feeding microstrip line on bottom copper layer.

Based on the above-mentioned equivalent transmission line, the optically frequency reconfigurable Vivaldi antenna is designed in the form of a tapered slot structure constructed using the copper with a thickness of 0.03 ${\text{mm}}$. The copper is printed on the front of a FR4 substrate with a size of $L_{S} \times H_{S}$, ${\upvarepsilon }_{r} = 4.6$ and $1.2\;{\text{ mm}}$ in thickness. The complete geometry of the proposed antenna as shown in Fig. 2 consists of three parts: top copper structure, FR4 substrate and bottom microstrip feed. The bottom copper layer of the substrate antenna is fitted with a microstrip line which ends with a broadband radial quarter wavelength stub related to the frequency band characteristics, thus forming a balun structure with the circular slotline cavity connected to the narrow end of the flare. The size of the flare is of practical significance to the radiation pattern of electromagnetic waves transmitted from the balun through the tapered slot. Detailed structure parameters are listed in Table 1.

**Table 1:** Detailed parameters of the antenna design.

Parameter	Value	Parameter	Value
${L}_{S}$	148.13 mm	${W}_{s1}$	1.08 mm
${L}_{S1}$	40.00 mm	${W}_{s2}$	5.07 mm
${L}_{S2}$	70.00 mm	${W}_{s3}$	15.84 mm
${L}_{S3}$	110.00 mm	${W}_{m}$	1.00 mm
${L}_{c}$	17.61 mm	${R}_{m}$	20.00 mm
${L}_{m}$	21.37 mm	${D}_{c}$	9.78 mm
${H}_{S}$	78.00 mm	$e$	2.00 mm
${H}_{m}$	28.69 mm	$g$	0.62 mm
${H}_{c}$	38.69 mm	${\theta }_{m}$	59.69°
${H}_{mc}$	11.12 mm	${\theta }_{mc}$	97.30°

The Vivaldi structure itself produces a wide operating band corresponding to the solt size. In order to reconfigure the resonate frequency of the antenna and exercise independent control over lower narrowband frequency, however, there are three photodiodes $P_{1}$, $P_{2}$, and $P_{3}$ integrated into different parts across the tapered slot with branch parallel structure on the antenna: $L_{s1} = 40\;{\text{ mm}}$, $L_{s2} = 70 \;{\text{mm}}$, $L_{s3} = 110\;{\text{ mm}}$.

**Figure 3:**
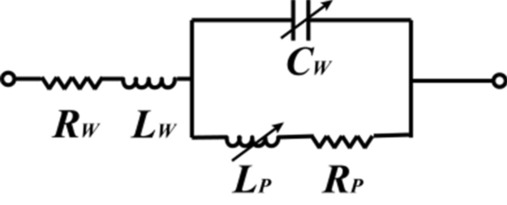
Equivalent circuit of the photodiode LXD1616R.

The photodiode has two working modes operating as a switch in the antenna structure: active and inactive. Referring to the property of the photodiode in^32^ and through the measurement of the photodiode, the equivalent circuit of photodiodes is constructed considering the impact of high frequency parasitic parameters, as illustrated respectively in Fig. 3. The photodiode acts as a capacitance $C_{w}$, a inductance $L_{w}$, a resistance $R_{w}$ composed in series, then another inductance $L_{p}$ and resistance $R_{p}$ in parallel with $C_{w}$. The most important property of the photodiode is that its impedance varies under different external illumination conditions. When the photodiode stays in the inactive state, the initial values of capacitance $C_{w}$ and resistances $R_{w} , R_{p}$ are 1.15 pF, 26 Ω, 10 KΩ. With a stimulus of white light over 400 lx, the values of $C_{w} ,\; R_{w} ,\; R_{p}$ change to 1.69 pF, 0.75 Ω, 0.1 Ω, respectively, and the photodiode switches to active state. The accurate parameter variation is listed in Table 2. The light switching component in this antenna design is taken as photodiode type LXD1616R, which has a frequency response of GHz level different from other photodiodes after actual measurement. However, for the frequency above 2 GHz, this photodiode shows a stable state of open circuit regardless of any illumination state.

**Table 2:** Parameter variation of the equivalent circuit.

Parameters in dark	Value	Parameters in light	Value
$C_{w}$	1.15 pF	$C_{w}$	1.69 pF
$L_{W}$	2.64 nH	$L_{W}$	2.64 nH
$L_{p}$	25.82 nH	$L_{p}$	25.82 nH
$R_{W}$	26 Ω	$R_{W}$	0.75 Ω
$R_{P}$	10 KΩ	$R_{P}$	0.1 Ω

The current on the Vivaldi antenna flows at the edge of the tapered slot, as a result of which the branches and photodiodes integrated to the slot have additional impedance attached to change the distribution of resonant current for the antenna, with special operating frequency band reconstructed. The position and amount of the branches and photodiodes are closely related to the variation of resonant frequency. Different branches can realize the current response of corresponding frequency, while the current only acts on the slot in the high frequency band, so an independent decoupling effect is realized. In case that the antenna continues to work at a higher non-adjustable frequency, there would be three resonant frequencies appearing under the influence of three photodiodes in design: $f_{1}$ = 300 MHz, $f_{2}$ = 677 MHz, $f_{3}$ = 1.02 GHz. There is a corresponding relationship existent between the working modes of three photodiodes and three resonant frequencies, which enables the antenna to switch between multiple frequencies controlled in a way that can make adjustment to the exact illumination conditions.

## Simulation

**Figure 4:**
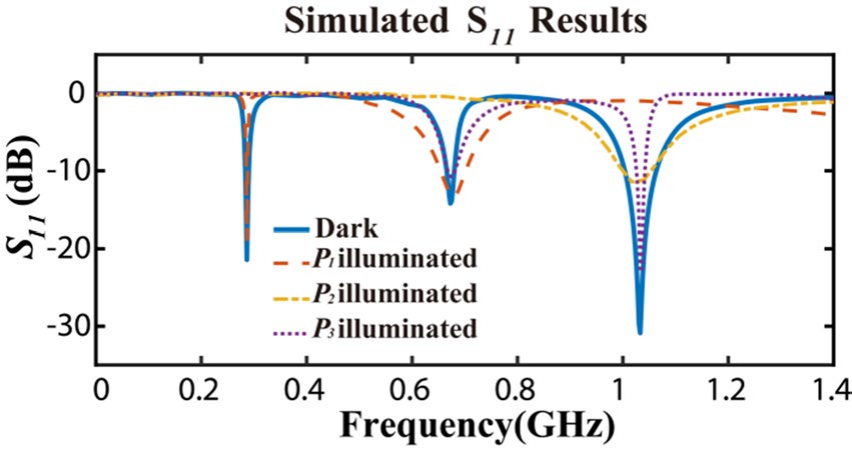
Simulation results of *S*_*11*_ parameters in different illumination states.

In consideration of the previous analysis, the simulation of the designed antenna integrated with photodiodes is carried out with the assistance of CST Microwave Studio. The simulation models of photodiodes in different illumination states with equivalent circuits are constructed to analyze the corresponding relationship between the working modes of photodiodes and the resonant frequencies of the proposed antenna. Figure 4 shows the simulation results of the *S*_*11*_ parameters. When the photodiode $P_{1}$ is left in the active state, which is under illumination condition, the resonant frequency $f_{3}$ ceases to maintain an working state while ${ }f_{1}$ and $f_{2}$ are out of influence. When the photodiode $P_{2}$ is activated, only $f_{3}$ remains working. When the photodiode $P_{3}$ is activated, the frequency $f_{1}$ is disturbed out of working. Table 3 shows the summary of relationship between working modes of three photodiodes and three reconfigurable frequencies, where Y represents the frequency remains working and N represents the frequency is out of working.

**Table 3:** Relationship between activated photodiodes and three reconfigurable working frequencies.

Activated photodiodes	${f}_{1}$ = 300 MHz	${f}_{2}$ = 677 MHz	${f}_{3}$ = 1.02 GHz
${P}_{1}$	Y	Y	N
${P}_{2}$	N	N	Y
${P}_{3}$	N	Y	Y
None	Y	Y	Y

**Figure 5:**
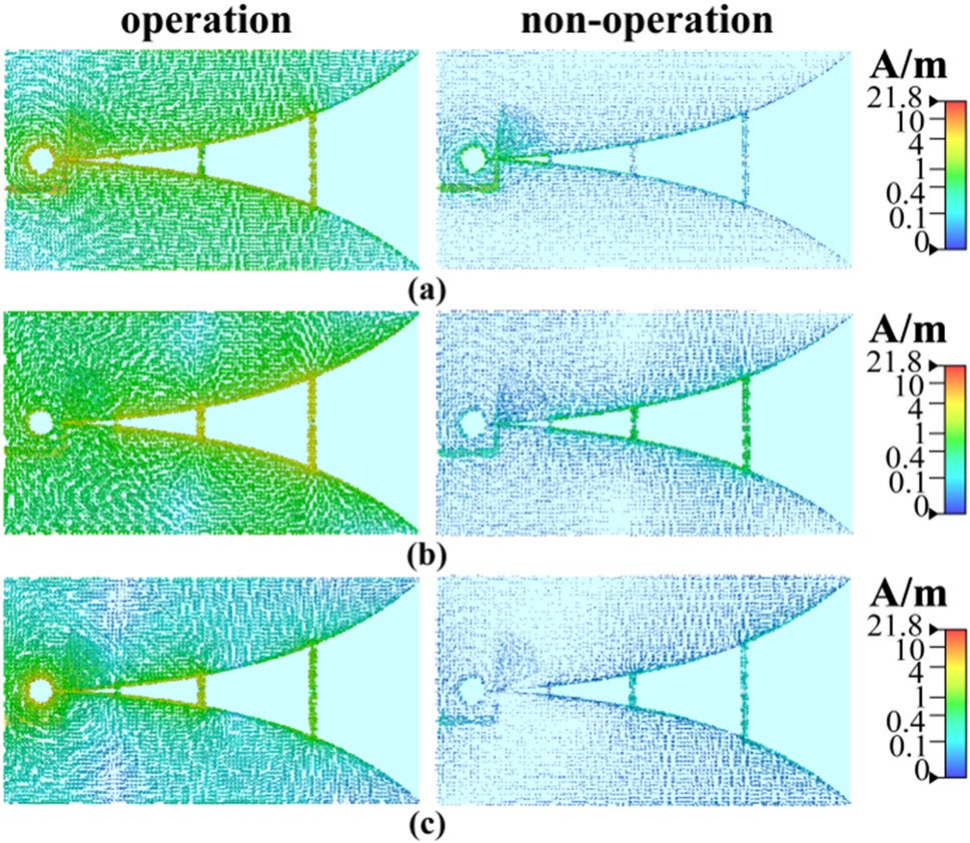
Simulation changes of the current distribution: (**a**) operation and non-operation states at 300 MHz; (**b**) operation and non-operation states at 677 MHz; (**c**) operation and non-operation states at 1.02 GHz.

For further analysis, the simulation diagrams of the current distribution at different frequencies are shown in the Fig. 5, from which it can be seen that the surface current varies significantly in different states due to the impact of illumination conditions. In particular, the branches at different parts play a significant role in different working frequencies.

**Figure 6:**
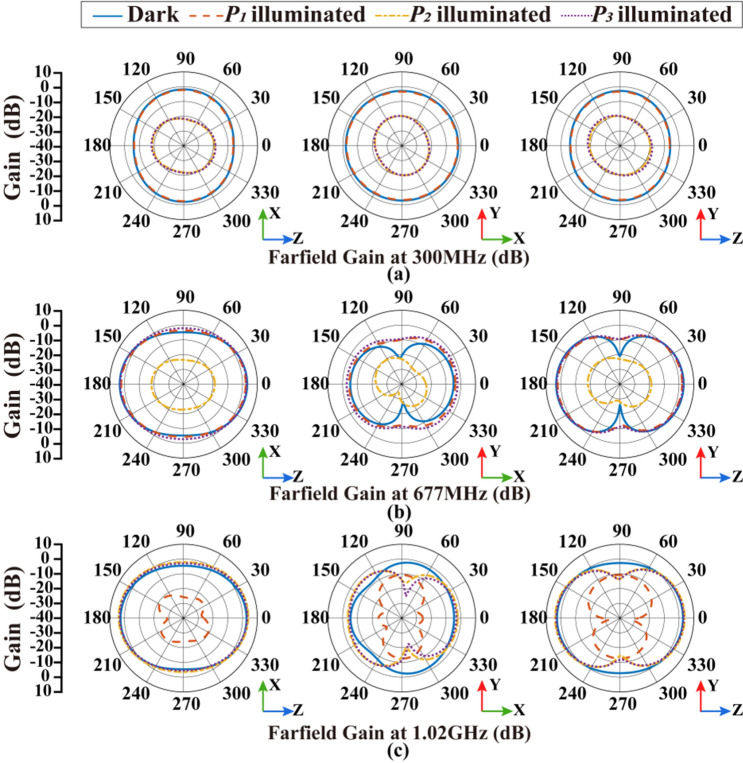
Simulation results of radiation patterns under different illumination conditions: (**a**) radiation pattern at 300 MHz; (**b**) radiation pattern at 677 MHz; (**c**) radiation pattern at 1.02 GHz.

The simulation of radiation patterns is also conducted under different illumination conditions, with the results shown in Fig. 6 and summarized in Table 4. There is a significant variation in the gain of the corresponding frequency when the photodiodes at different parts are individually activated. The frequency $f_{1}$ (300 MHz) is easy to be affected by the activation of photodiode $P_{2}$ or $P_{3}$, and the maximum gain declines from − 1.84 to − 17.61 dB. The antenna gain at $f_{2} ($677 MHz) can be adjusted by activating photodiode $P_{2}$ from the maximum value 3.14 to − 18.15 dB. The frequency $f_{3}$ (1.02 GHz) is affected by activating photodiode $P_{1}$, which means the maximum gain changes from 3.91 to − 10.78 dB. Moreover, under illumination condition, the efficiency of the antenna at inoperative frequencies decreases significantly. The maximum gain difference caused by the illumination conditions can reach up to 21.29 dB, indicating the effect of photodiode switches on the resonant frequency of the antenna. The front-to-back ratios (FBRs) of the maximum radiation direction are 0.21 dB, 0.04 dB, and 0.03 dB at 300 MHz, 677 MHz, and 1020 MHz, respectively, which also shows an omnidirectional radiation performance of the proposed antenna at low frequencies.

**Table 4:** Simulated variation in radiation performance under different illumination condition.

Frequency (MHz)	Illuminated photodiodes	Maximum gain (dB)	Efficiency %	FBR (dB)	Max gain variation (dB)	Bandwidth (MHz)
$f_{1}$ = 300	$P_{1}$	− 1.88	72.96	0.21	15.77	8
	$P_{2}$	− 17.61	1.028			
	$P_{3}$	− 17.61	2.598			
	None	− 1.84	82.78			
$f_{2}$ = 677	$P_{1}$	2.71	69.02	0.04	21.29	16
	$P_{2}$	− 18.15	12.13			
	$P_{3}$	2.75	86.60			
	None	3.14	92.45			
$f_{3} = 1020$	$P_{1}$	− 10.78	16.20	0.03	14.69	56
	$P_{2}$	3.91	89.96			
	$P_{3}$	3.68	90.34			
	None	3.32	96.60			

## Measurement results

**Figure 7:**
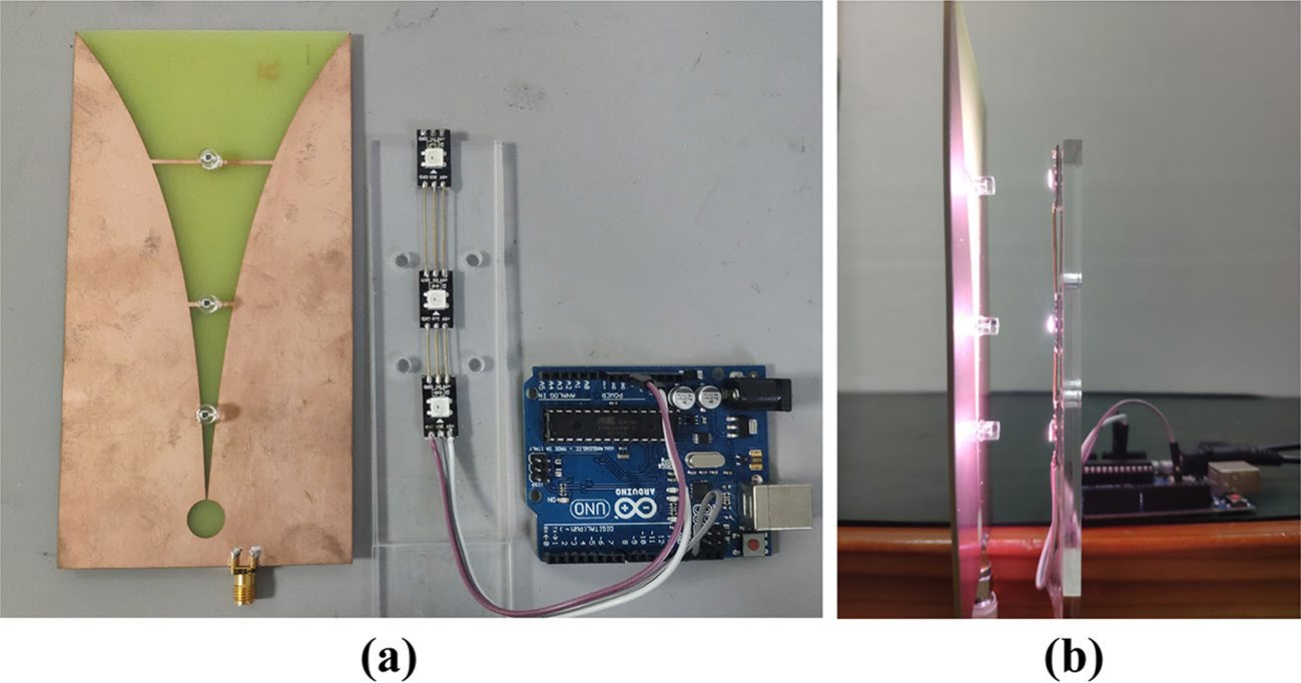
Experimental photos: (**a**) the Antenna, LEDs, and Arduino; (**b**) the experimental setup of antenna with LED lighting illumination.

An optically frequency reconfigurable Vivaldi antenna is produced in line with the design proposed in section “Antenna design”. Moreover, a programmable illumination control device is developed in order to alter the frequency response of the tunable Vivaldi antenna. Figure 7 shows the full details of the antenna design and frequency configurable system, including the proposed antenna, WS2812B LEDs and Arduino UNO. The Arduino UNO is applied to gain control on the antenna working condition with the setting of LEDs brightness. Through the illumination of different LEDs on corresponding photodiodes, the working modes of three photodiodes are changed. In doing so, the antenna can be switched between three working frequencies. In the course of experiment operation, the LEDs are set to continuously emit white light over 800 lx mixed by red, green, and blue lights with a wavelength λ of 700.00 nm, 546.10 nm, 438.8 nm, respectively. The distance between the photodiode and corresponding LED is 25 mm. Conversely, when the LED is switched off, the brightness of ambient light drops below 200 lx in the indoor sheltered environment. The illumination of LED on the other photodiodes at a horizontal distance of 30 mm is 160 lx, 84 lx at 40 mm and less than 5 lx at 70 mm.

**Figure 8:**
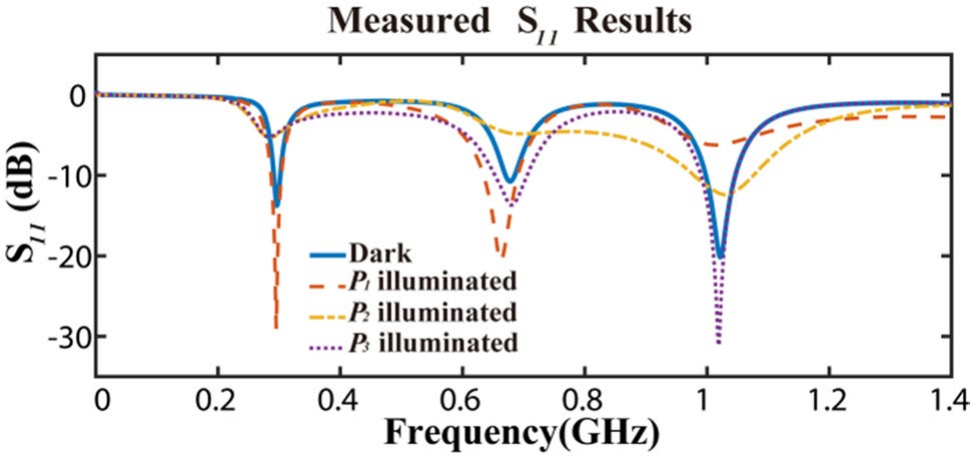
Measured results of *S*_*11*_ parameters.

**Figure 9:**
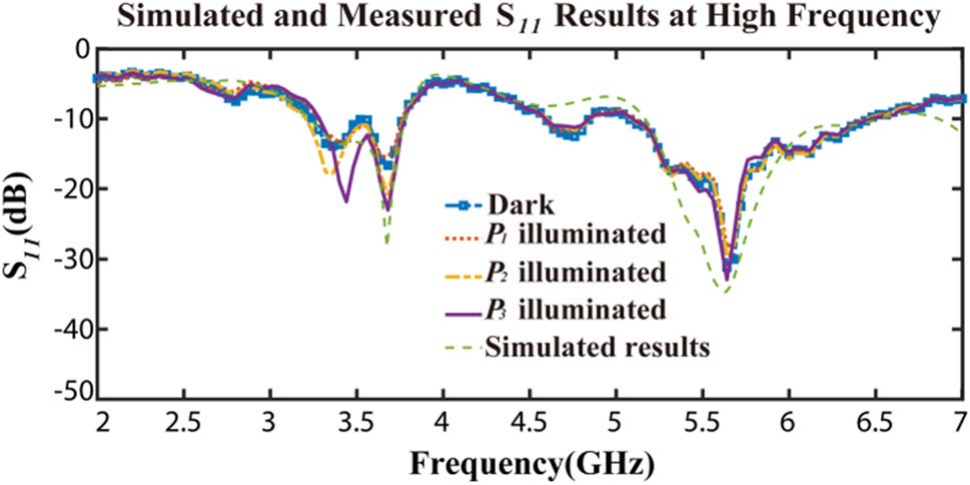
Simulated and measured results of *S*_*11*_ parameters at high frequency.

The measurement of the proposed antenna is performed and the *S*_*11*_ results shown in Fig. 8 are highly consistent with the simulation results as mentioned above. The working bandwidths are 12 MHz (293–305 MHz), 15 MHz (671–686 MHz), 50 MHz (997–1047 MHz) frequencies below − 10 dB. In contrast, according to the measurement results of *S*_*11*_ parameter in the high frequency range, there are stable working bands at 3.2–3.8 GHz and 5.1–6.5 GHz regardless of illumination, as shown in Fig. 9.

**Figure 10:**
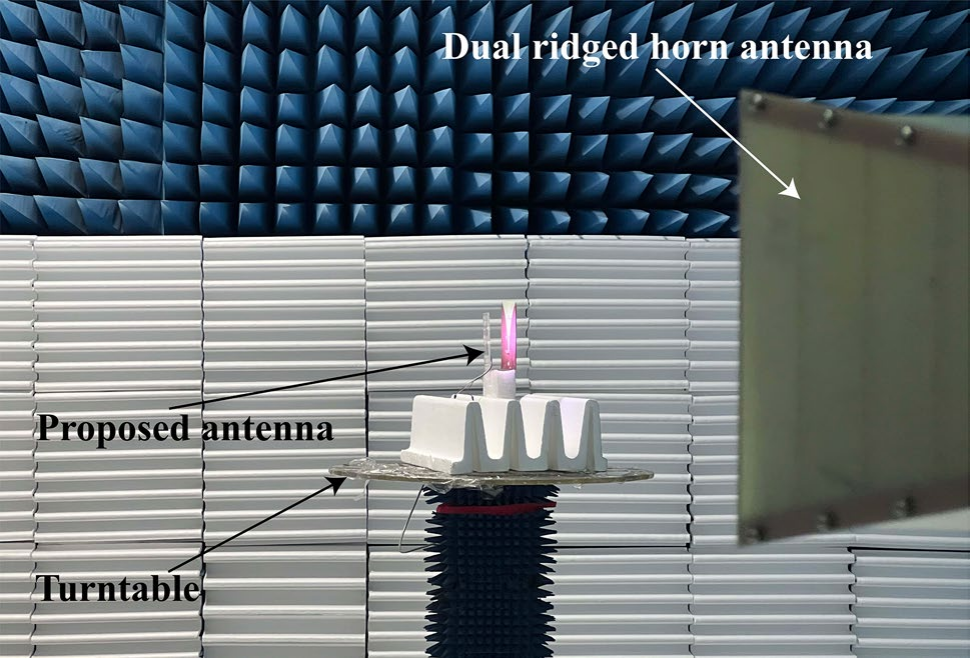
The measurement system in microwave anechoic chamber.

**Figure 11:**
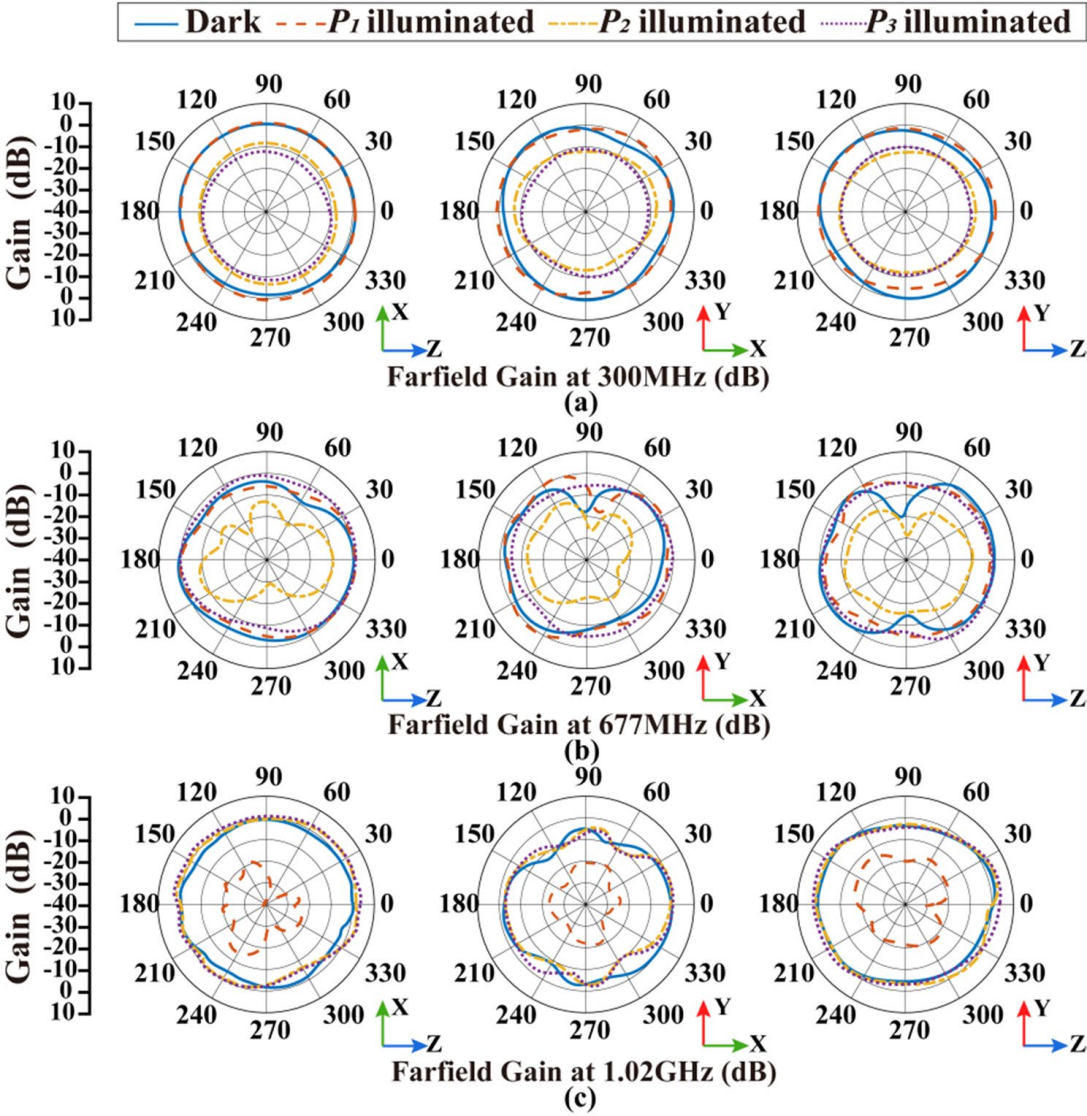
Measured results of radiation patterns under different illumination conditions: (**a**) radiation pattern at 300 MHz, (**b**) radiation pattern at 677 MHz, (**c**) radiation pattern at 1.02 GHz.

The radiation patterns of the antenna at all available frequencies are measured in microwave anechoic chamber, as shown in Fig. 10. The measurement system consists of the proposed antenna, a dual ridged horn antenna (HD-1018DRHA), the turntable and network analyzer (R&SZNB20). Figure 11 shows the measured results which are consistent with the simulation results shown in Fig. 6. The maximum of antenna gain under non-illumination condition at $f_{1}$ (300 MHz), $f_{2}$ (677 MHz), $f_{3}$ (1.02 GHz) is 0.91 dB, 1.69 dB, 2.96 dB, respectively, while the variation in illumination states is 6.82 dB, 9.93 dB, 17.13 dB, respectively, as summarized in Table 5. The maximum value of the antenna gain under non-illumination at $f_{1}$ (300 MHz) is 0.91 dB, which changes to − 5.91 dB under the illumination on photodiode $P_{2}$ or $P_{3}$. When the designed antenna works at $f_{2}$ (677 MHz), the maximum of the antenna gain without illumination reaches 1.69 dB, and the value in the presence of illumination on photodiode $P_{2}$ is − 8.24 dB. The maximum of the antenna gain under non-illumination at $f_{3}$ (1.02 GHz) is 2.96 dB, which is changed by 17.13 dB to − 14.17 dB in the presence of illumination on photodiode $P_{1}$. The measurement results prove that this antenna works efficiently with control on the illumination conditions and shows a significant gain variation at low frequencies. In addition, the FBR of the antenna under operating state is 0.33 dB, 0.81 dB, and 0.52 dB, respectively at $f_{1}$ (300 MHz), $f_{2}$ (677 MHz), $f_{3}$ (1.02 GHz). It means these radiation patterns are all omnidirectional. Generally conventional Vivaldi antenna with such a small size is unable to work at low frequency below its size limit, but attributed for extra constructed current circuit loops this new reconfigured antenna structure has optically switchable narrow bands.

**Table 5:** Measured variation in radiation performance under different illumination condition.

Frequency (MHz)	Illuminated photodiodes	Maximum gain (dB)	FBR (dB)	Max gain variation (dB)	Bandwidth (MHz)
${f}_{1}$ = 300	${P}_{1}$	0.91	0.33	6.82	12
	${P}_{2}$	− 5.91			
	${P}_{3}$	− 7.61			
	None	0.34			
${f}_{2}$ = 677	${P}_{1}$	0.62	0.81	9.93	15
	${P}_{2}$	− 8.24			
	${P}_{3}$	0.88			
	None	1.69			
${f}_{3}=1020$	${P}_{1}$	− 14.17	0.52	17.13	50
	${P}_{2}$	2.05			
	${P}_{3}$	2.96			
	None	1.33			

**Figure 12:**
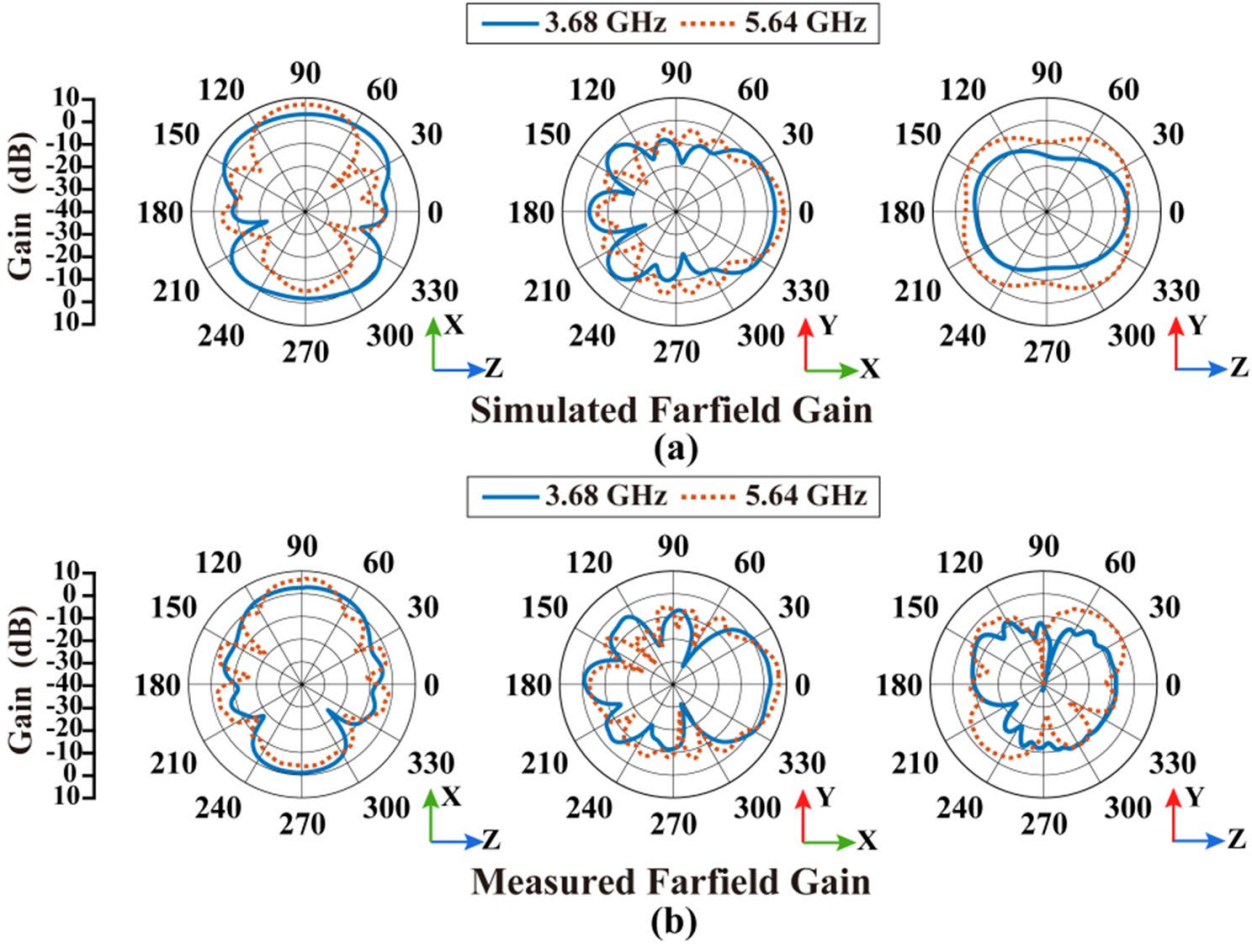
Simulated and measured results of radiation patterns at high frequencies: (**a**) simulated results at 3.68 GHz and 5.64 GHz, (**b**) measured results at 3.68 GHz and 5.64 GHz.

The radiation patterns of the antenna at higher frequencies above 2 GHz are also simulated and measured, described in Fig. 12. Representative operating frequencies of 3.68 GHz and 5.64 GHz were selected. The measurement results in Fig. 12b are consistent with the simulation results in Fig. 12a. The maximum radiation gain of 3.68 GHz is up to 3.55 dB, while 5.64 GHz is up to 7.51 dB. It shows that the proposed antenna still maintains good characteristics at high frequencies corresponding to the antenna size. The stable working bands can be kept from illumination interference beyond the frequency range of impedance variation response of photodiodes over 2 GHz. According to measurement results, the FBR at 3.68 GHz and 5.64 GHz is 4.31 dB and 11.23 dB. It also reflects the antenna has directional characteristics at high frequencies while it has omnidirectional radiation pattern at low frequencies.

The comparison between the proposed antenna and other reconfigurable antennas are listed in Table 6. Compared to the proposed antenna, the referred antennas electronically controlled by diodes with biasing circuits^4,8^ are unable to realize the gain variation of the center frequency without interference in other bands, which are suitable for implementing the frequency shift and bandwidth change. Meanwhile, compared to other optically controlled antennas^21,28,29^, the proposed antenna has greater gain variation and more reconfigurable bands. In addition, a comparison with^28^ shows that the designed antenna is able to work at lower frequencies with a smaller size. In general, the proposed antenna combines the adjustable low-frequency omnidirectional radiation and high-frequency directional stable radiation, besides the decoupling effect of independent operation between different operating bands of the antenna is also realized, which is not found in other designs.

**Table 6:** Comparison between other reconfigurable antennas and the proposed antenna.

Antenna	Size	Min frequency (GHz)	Switches	Max gain variation (dB)	Center frequency shift (GHz)	Reconfigured bands
Proposed antenna	148.13 × 78 × 1.23 mm^3^	0.30	3 Photodiodes	17.13	0.006	3
^4^	50 × 50 mm^2^	2.89	4 PIN diodes	0.10	2.17	1
^8^	40 × 30 × 0.10 mm^3^	1.82	2 PIN diodes	3.16	1.17	4
^21^	30 × 30 × 1.60 mm^3^	11.00	2 Silicon switches	5.12	0.01	1
^28^	200 × 150 × 1.63 mm^3^	0.74	2 Photodiodes	0.9	0.03	1
^29^	131 × 21.5 × 1.60 mm^3^	1.99	4 Photodiodes	6.1	0.007	2

## Conclusion

In this paper, a three-frequency optically switched antenna developed on the basis of Vivaldi structure is proposed. With the integration of three parallel branches composed of LXD1616R photodiodes at different parts on the antenna radiator, the antenna is capable to work at the frequencies of 300 MHz, 677 MHz, 1.02 GHz through the switching of LED illumination. The maximum gain variation reaches up to 17.13 dB as compared to different illumination modes. In the absence of direct sunlight, the antenna can be controlled by LEDs or lasers in a long distance. What’s more, this antenna works steadily at higher frequencies regardless of illumination interference, while its maximum radiation gain could reach 3.55 dB and 7.51 dB at 3.68 GHz and 5.64 GHz. This variety of optically reconfigurable Vivaldi antenna design can be applied to transmit, receive and shield three low frequency signals in wireless communication by imposing optical control. The two bands of high frequency out of influence of light have a potential of radar detection. Considering its multi frequency radiation and adjustable characteristics, it will be of massive application value in communication and radar integration.

## Data Availability

The data produced and analyzed during the current study are available from the corresponding author on reasonable request.
